# Anti-RBD IgG antibodies and neutralizing antibody levels after the second BNT162b2 dose in patients with plasma cell disorders

**DOI:** 10.1371/journal.pone.0284925

**Published:** 2023-05-01

**Authors:** Hila Magen, Abraham Avigdor, Lee Nevo, Shalev Fried, Amit Gibori, Einav G. Levin, Yaniv Lustig, Eden Shkury, Galia Rahav

**Affiliations:** 1 Division of Hematology and Bone-Marrow Transplantation, Sheba Medical Center, Ramat Gan, Israel; 2 Sackler Faculty of Medicine, Tel-Aviv University, Tel Aviv, Israel; 3 The Infection Prevention and Control Unit, Sheba Medical Center, Ramat Gan, Israel; 4 Central Virology Laboratory, Israel Ministry of Health and Sheba Medical Center, Ramat Gan, Israel; 5 Infectious Disease Unit and Laboratory, Sheba Medical Center, Ramat Gan, Israel; Regional Medical Research Centre Bhubaneswar, INDIA

## Abstract

Patients with plasma cell disorders (PCD) are at an increased risk for severe morbidity and mortality due to COVID-19. Recent data have suggested that patients with hematological malignancies, including those with PCD, have suboptimal antibody response to COVID-19 vaccination. We compared the antibody titers of 213 patients with PCD to those of 213 immunocompetent healthcare workers after the second vaccine dose of the BNT162b2 mRNA vaccine. Blood samples were taken 2–4 weeks after the second vaccination and analyzed for anti-receptor binding-domain immunoglobulin G (RBD-IgG) antibodies and neutralizing antibodies (NA). At a median of 20 days after the second vaccine dose, 172 patients (80.8%) developed anti-RBD-IgG antibodies with a geometric mean titer (GMT) of 2.7 (95% confidence interval [CI], 2.4–3.1). In the control group 210 (98.9%) developed anti-RBD-IgG antibodies after a median of 21 days, with a GMT of 5.17 (95%CI, 4.8–5.6), p<0.0001. NA were observed in 151 patients with MM (70.9%) and in 210 controls (98.9%). The GMT of NA in patients with MM and controls was 84.4 (95% CI, 59.0–120.6), and 420.2 (95% CI, 341.4–517.1), respectively (p<0.0001). Multivariable logistic regression revealed that the number of prior therapy lines and age were significant predictors of poor humoral response among patients with MM. Injection site reaction, headache and fatigue were the most common adverse events after vaccination. Adverse events were less common in patients with MM than in controls. In conclusion, a significant percentage of patients with MM developed protecting NA to the BNT162b2 mRNA vaccine, which appears to be safe in this patient population.

## Introduction

Israel was one of the first countries to start a national vaccination campaign against severe acute respiratory syndrome coronavirus 2 (SARS-CoV-2) shortly after the Pfizer-BioNTech mRNA vaccine (BNT162b2) received emergency use authorization by the United States Food and Drug Administration in December 2020 [1]. The vaccination campaign began during the third wave of COVID-19 in the country. Initially, the vaccine was administered to front-line healthcare workers (HCW), people aged 60 years and over, nursing home residents and other people at high risk due to serious medical conditions [1].

Cancer patients, including those with hematological malignancies [2, 3] such as multiple myeloma (MM) and other plasma cell disorders (PCD) [4], have a higher risk for a severe outcome following infection with SARS-CoV-2 [5–7].

Clinical trials with the BNT162b2 mRNA vaccine did not include immunosuppressed subjects [8, 9]. The Israeli Ministry of Health approved the BNT162b2 vaccine for patients treated with immunosuppressive therapy or biological response modifiers associated with any malignancy, individuals who had solid organ transplantation, stem cell transplantation or splenectomy, and individuals with primary immune-deficiency or with human immunodeficiency virus [1].

We evaluated the safety and humoral response, namely, the levels of neutralizing antibodies (NA) and anti-receptor-binding domain (RBD) IgG antibodies following vaccinations with the Pfizer-BioNTech BNT162b2 mRNA vaccine among 213 patients with PCD, including MM, and 213 immunocompetent HCW. We also examined if the stage of PCD or type of therapy administered affected the observed humoral response.

## Methods

### Study design and population

Following the authorization of the BNT162b2 mRNA vaccine in Israel, we advised all patients with PCD treated at our medical center to get vaccinated according to standard guidelines [10].

In the first three months thereafter, we offered all patients who were scheduled for routine clinic visits the opportunity to participate in a prospective study evaluating antibody response, clinical efficacy and adverse events related to the vaccine. Two-hundred and thirteen adult patients with MM (>18 years) who consented to be vaccinated and to participate in the study, and for whom there was a serology test result 2–4 weeks after the second dose of the vaccine, were included in the study. Patients who had recovered from COVID-19 or had active COVID-19 at the time of the vaccination or up to seven days after receiving the second vaccine dose were excluded. Active COVID-19 infection was diagnosed according to disease symptoms and confirmed using a positive quantitative real-time polymerase chain reaction (qRT-PCR) test.

The control group comprised 213 HCW at Sheba Medical Center (Ramat Gan, Israel) who were tested for antibody response 2–4 weeks after the second vaccine.

Written informed consent was obtained from all participants. The protocol and informed consent were approved by the institutional review board (7982-20-SMC for patients with MM and 8008-20-SMC for immunocompetent HCW).

### Data extraction

Relevant clinical data were retrieved from electronic medical records and included age, gender, comorbidities (hypertension, ischemic heart disease, diabetes mellitus, chronic obstructive pulmonary disease, other malignancies). Disease history data included date of PCD diagnosis and MM International Staging System (ISS) score. The start dates of treatment lines, therapy combinations, and response to therapy were also retrieved. Anti-MM therapy included immunomodulatory drugs (IMiDs): lenalidomide, pomalidomide; proteasome inhibitors (PIs): bortezomic, carfilzomib, ixazomib; the anti-CD38 monoclonal antibody daratumumab; and corticosteroids (mostly dexamethasone). Laboratory data included blood counts and biochemistry (electrolytes, lactate dehydrogenase, renal and hepatic function tests), immunoglobulin levels (IgG, IgA, IgM), immunoelectrophoresis, immunofixation and free light chains (FLC) assay. Immunoparesis was defined as one or more uninvolved immunoglobulins below the normal levels (complete immunoparesis was defined as suppression of all 3 immunoglobulins; partial immunoparesis was defined as suppression of 1–2 immunoglobulins). Response to MM treatments was determined according to the International Myeloma Working Group (IMWG) criteria [11].

### Safety

Adverse events (AEs) that occurred within 30 days after each vaccine dose were recorded using specific questionnaires. Patients were instructed to report any suspected local (pain, erythema, swelling at the injection site, and lymphadenopathy) or systemic reactions (fever, fatigue, headache, myalgia, chills, nausea, vomiting, or paresthesia), and were actively screened for any other systemic or local complaints.

### Serology assays

Samples from participants were tested with an enzyme-linked immunosorbent assay (ELISA) that detects anti-RBD IgG antibodies of SARS-CoV-2. Titers >1.1 arbitrary units (AU)/mL were defined as positive. A SARS-CoV-2 pseudo-virus neutralization assay was performed using a propagation-competent vesicular stomatitis virus (VSV) spike, similar to that previously published [12], which was kindly provided by Gert Zimmer (University of Bern, Bern, Switzerland). Sera not capable of reducing viral replication by 50% at a 1–8 dilution or below were considered non-neutralizing.

### Statistical methods

Data were analyzed using the SAS ® version 9.4 (SAS Institute, Cary North Carolina). Continuous variables were summarized as means with standard deviation (SD) or median and range (minimum-maximum), and categorical variables were summarized as numbers and percentages. Titers were summarized as geometric mean (GMT) with 95% confidence interval (CI). Continuous variables were compared using t-test and categorical variables were compared using chi-squared test. Multivariable logistic regression analysis was used to determine the influence of age, co-morbidities (cardiovascular disease and chronic obstructive pulmonary disease), past or present treatment with daratumumab, treatment with anti-MM treatment at the time of vaccination, and the number of therapy lines on the magnitude of response to the second dose of the vaccine. All tests were two-tailed, and a p value of 5% or less was considered statistically significant.

## Results

### Baseline characteristics

The clinical characteristics of the MM cohort are summarized in Table 1. Most patients (87.3%) had MM (including amyloidosis), 10.4% had monoclonal gammopathy of undetermined significance or smoldering myeloma and 2.4% had other PCD. Over half of patients (56.1%) had partial immunoparesis and 13.6% had complete immunoparesis. At the time of vaccination, 73.3% of patients were treated for MM: 28.3% with daratumumab, 17.5% with an IMiD and 17.5% with a PI. Corticosteroids, most commonly dexamethasone 10–40 mg per week, were an integral part of all MM protocols.

**Table 1:** PCD cohort characteristics.

	Multiple Myeloma N = 213
**Monoclonal disorder stage, n (%)**	
Multiple myeloma (including amyloidosis)	186 (87.3%)
Monoclonal gammopathy of undetermined significance + smoldering multiple myeloma	22 (10.4%)
Other	5 (2.4%)
**International Staging System score, n (%)**	
1	90 (42.2%)
2	40 (18.8%)
3	38 (17.8%)
Unknown	45 (21.1%)^a^
**Immunoparesis, n (%)**	
Absent	48 (22.6%)
Partial (suppression of 1–2 immunoglobulins)	119 (56.1%)
Complete (suppression of all 3 immunoglobulins)	29 (13.7%)
Unknown	16 (7.6%)
**Autologous bone marrow transplantation, n (%)**	126 (59.2%)
**Treatment at vaccination, n (%)**	156 (73.2%)
Corticosteroids	213 (100%)
Daratumumab-based therapy	60 (28.3%)
Immunomodulatory drug	37 (17.5%)
Proteasome inhibitors	37 (17.5%)
Immunomodulatory drug + proteosome inhibitor	10 (4.7%)
Other^b^	12 (7.7%)
**Daratumumab-based therapy (at any stage), n (%)**	97 (45.5%)
**Intravenous immunoglobulin supportive treatment at the time of vaccination, n (%)**	23 (10.9%)
**Response status**^**c**^ **at first dose of vaccine, n (%)**	
Complete response (CR) + stringent CR	55 (25.8%)
Very good partial response + partial response	97 (45.5%)
Stable disease	35 (13.4%)
Progressive disease	15 (7.0%)
Not evaluated^d^	11 (5.2%)

^a^ Twenty-two patients with smoldering multiple myeloma/monoclonal gammopathy of undetermined significance, 20 patients with multiple myeloma, one patient each with POEMS syndrome, Waldenstrom macroglobulinemia, and amyloidosis.

^b^ Selinexor; belantamab mafodotin; melphalan and bendamustin

^c^ Response to MM treatments was determined according to International Myeloma Working Group (IMWG) criteria: **stringent complete response (sCR)** = below plus normal free light chain (FLC) ratio and absence of clonal cells in bone marrow by immunohistochemistry or immunofluorescence; **complete response** (CR (= negative immunofixation on the serum and urine and disappearance of any soft tissue plasmacytomas and <5% plasma cells in bone marrow; **very good partial response (VGPR)** = serum and urine M-protein detectable by immunofixation but not on electrophoresis or >90% reduction in serum M-protein plus urine M-protein level <100 mg/24 hours; **partial response (PR)** = >50% reduction of serum M-protein and reduction in 24 hours urinary M-protein by >90% or to < 200 mg/24 hours. If the serum and urine M-protein are unmeasurable, a >50% decrease in the difference between involved and uninvolved FLC levels is required in place of the M-protein criteria. If serum and urine M-protein are not measurable, and serum free light assay is also not measureable, >50% reduction in plasma cells is required in place of M-protein, provided baseline bone marrow plasma cell percentage was > 30%. In addition to the above listed criteria, if present at baseline, a >50% reduction in the size of soft tissue plasmacytomas is also required; **progressive disease** = Increase of >25% from lowest response value in any one or more of the following: serum M-component and/or urine M-component (in patients without measurable serum and urine M-protein, the level difference between involved and uninvolved FLC levels is measured), and/or bone marrow plasma cell percentage (the absolute percentage must be >10%), and/or definite development of new bone lesions or soft tissue plasmacytomas or definite increase in the size of existing bone lesions or soft tissue plasmacytomas; and/or development of hypercalcaemia (corrected serum calcium >11.5 mg/dL or 2.65 mmol/L) that can be attributed solely to the plasma cell proliferative disorder; **stable disease** = not meeting criteria for CR, VGPR, PR, or progressive disease.

^d^Subjects were never treated for their monoclonal gammopathy.

The demographic characteristics and comorbidities of the PCD cohort and the immunocompetent HCW control group are compared in Table 2. The median age of the study population was 67 years (minimum-maximum range 35–91) and 59.6% were male. The control group and the PCD group has similar demographic characteristics as well similar serology timing after the second vaccine. The PCD cohort had statistically significant higher rates of cardiac disease (20.7% vs. 10.3%, p = 0.002) and chronic pulmonary disease (8.5% vs. 1.9%, p = 0.002) compared to the control group.

**Table 2:** Clinical and demographic characteristics of the study population.

Variable	Plasma cell disorders N = 213	Controls N = 213	p value
**Median age, years (range)**	67 (37–91)	67 (35–82)	0.58
**Sex, n (%)**			
Male	127 (59.6%)	127 (59.6%)	1.00
Female	86 (40.4%)	86 (40.4%)	
**Comorbidities/risk factors, n (%)**			
Hypertension	79 (37.1%)	75 (35.2%)	0.61
Diabetes mellitus	34 (16.0%)	25 (11.7%)	0.19
Cardiac disease	44 (20.7%)	22 (10.3%)	**0.002**
Chronic pulmonary disease	18 (8.5%)	4 (1.9%)	**0.002**
**Median number of days from second vaccine dose to serology**	20	21	0.06
**Humoral response, GMT (95% CI)**			
Neutralizing antibodies	84.4 (59.0–120.6)	420.2 (341.4–517.1)	**<0.0001**
Receptor binding domain IgG	2.7 (2.4–3.1)	5.2 (4.8–5.6)	**<0.0001**

CI = confidence interval, GMT = geometric mean titer, IgG = immunoglobulin G, SD = standard deviation

### Humoral response

**Fig 1:**
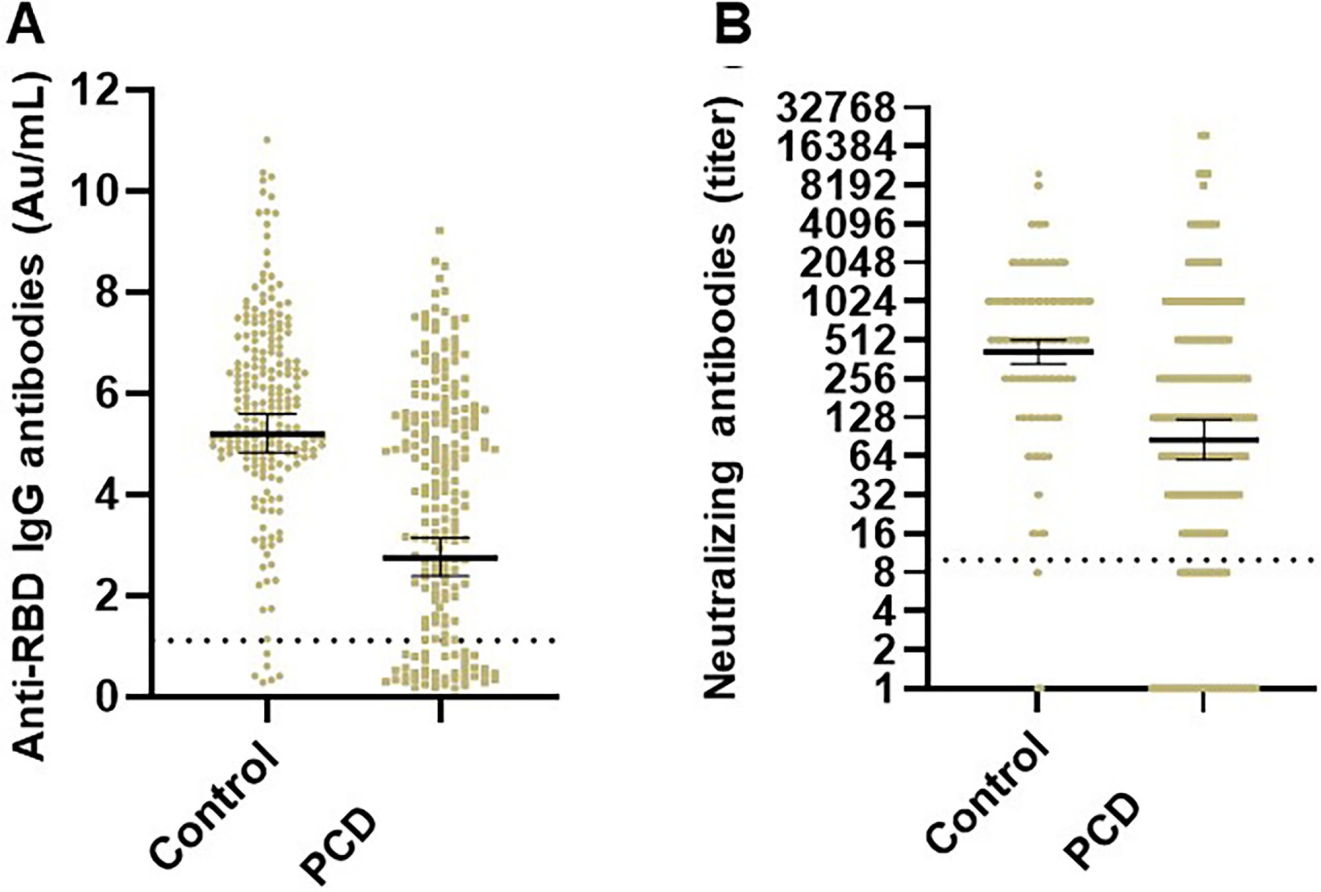
Comparison of anti-RBD IgG antibody levels (A) and neutralizing antibody levels (B) in immunocompetent healthcare workers and patients with plasma cell disorders (PCD). The black horizontal lines indicate mean and standard deviation.

**Fig 2:**
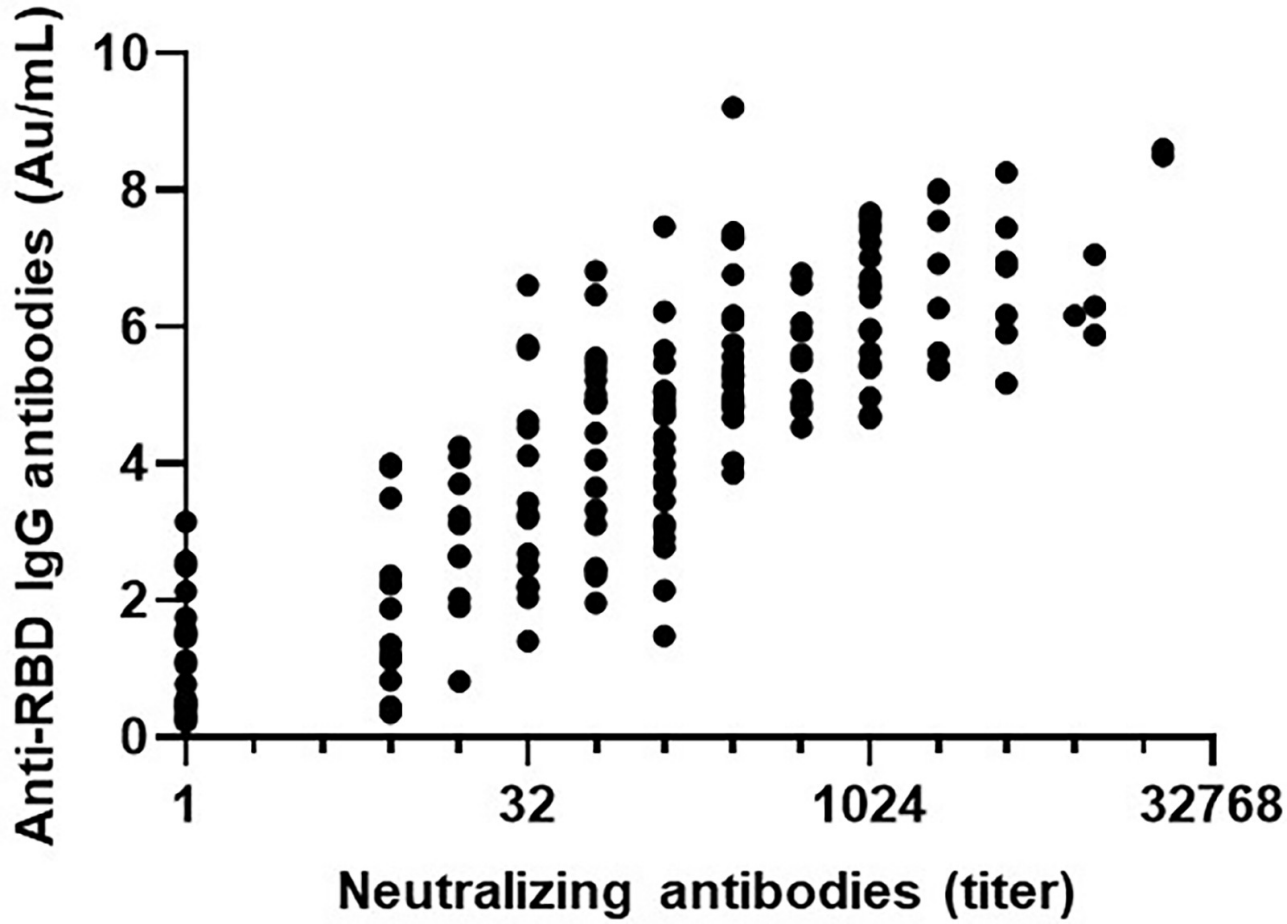
Correlation between anti-RBD IgG antibody levels and neutralizing antibody levels in patients with plasma cell disorders (PCD).

At a median of 20 days after receiving the second vaccine dose, 172 patients with PCD (80.8%) developed anti-RBD IgG antibodies with a GMT of 2.7 AU/mL (95% CI, 2.4–3.1). In the control group 210/213 individuals (98.9%) developed anti-RBD IgG antibodies at a median of 21 days after the second vaccine dose, with a GMT of 5.2 AU/mL (95% CI 4.8–5.6), p<0.0001. The NA GMT in PCD patients and controls was 84.4 (95% CI 59.0–120.6) and 420.2 (95% CI 341.4–517.1), respectively (p<0.0001), (Table 2 and Fig 1). As shown in Fig 2, a strong correlation was observed between anti-RBD IgG antibody levels and NA levels (r = 0.827 [95% CI, 0.773–0.868], p<0.0001).

Multivariable logistic regression analysis (Table 3) showed that a higher number of prior therapy lines and older age predicted lower humoral immune response (odds ratio [OR] = 1.52 [95% CI 1.09–2.10], p = 0.01 and OR = 1.04 [95% CI 1.00–1.09), p = 0.05, respectively).

**Table 3:** Multivariable regression analysis.

Variable	No neutralizing antibody response			No anti-RBD IgG antibody response		
	Odds ratio	(95% CI)	p value	Odds ratio	(95% CI)	p value
**Model 1**						
Age	1.04	(1.00–1.09)	**0.05**	1.04	(1.00–1.08)	0.07
Number of prior therapy lines	1.52	(1.09–2.10)	**0.01**	1.14	(0.85–1.53)	0.38
Treatment with daratumumab (ever)	0.84	(0.18–3.95)	0.82	0.49	(0.11–2.10)	0.33
Treatment with daratumumab at vaccination (vs. no treatment)	3.77	(0.68–20.98)	0.36	3.69	(0.67–20.37)	0.79
Treatment with IMiD + PI at vaccination (vs. no treatment)	4.01	(0.55–29.11)	0.45	7.64	(1.22–47.95)	0.20
Treatment with IMiD at vaccination (vs. no treatment)	1.84	(0.40–8.49)	0.70	2.31	(0.48–11.17)	0.50
Treatment with PI at vaccination (vs. no treatment)	1.12	(0.21–5.89)	0.19	1.89	(0.37–9.81)	0.27
Treatment with another regimen at vaccination* (vs. no treatment)	4.14	(0.38–45.12)	0.48	8.93	(1.16–68.56)	0.12
Cardiac disease (vs. no cardiac disease)	0.62	(0.22–1.72)	0.36	1.00	(0.39–2.60)	1.00
Chronic pulmonary disease (vs. no pulmonary disease)	1.22	(0.26–5.67)	0.80	0.88	(0.24–3.25)	0.85

*Selinexor; belantamab mafodotin; melphalan and bendamustin

CI = confidence interval, IMiD = immunomodulatory drug, PI = proteosome inhibitor, RBD IgG = receptor-binding domain immunoglobulin G

### Vaccination safety in MM patients

No vaccine-related serious AEs or allergic responses were observed among the study population. Local AEs were local pain, erythema, lymphadenopathy, or swelling at the injection site. Systemic AEs were fever, chills, fatigue, headaches, myalgia, paresthesia, nausea or vomiting.

The frequencies of local and systemic AEs were lower in patients with PCD compared to the control population; this difference was more pronounced and statistically significantly different after the second vaccine dose (p = 0.04, Table 4). The most common local reaction was injection-site pain, which was mild in most cases and subsided within 24 hours. Systemic AEs included mostly fatigue and headache. The frequency of systemic AEs was higher in both groups following administration of the second vaccine (18.3% vs. 15.5% in MM patients and 54.9% vs. 21.1% in controls).

**Table 4:** Adverse events type following the first and second vaccination with the BNT162B2 vaccine.

Adverse Event type	MM N = 213 n (%)	Controls N = 213 n (%)	P value
**First vaccination**			
Local (any)	102 (47.9)	155 (72.8)	0.69
Systemic (any)	33 (15.5)	45 (21.1)	
**Second vaccination**			
Local (any)	94 (44.1)	174 (81.7)	**0.04**
Systemic (any)	39 (18.3)	117 (54.9)	

p value was calculated using Fischer’s exact text

## Discussion

Since the authorization of the BNT162b2 vaccine, a growing number of studies have reported reduced responses in patients with hematologic malignancies [13–21]. We examined the humoral response to the BNT162b2 vaccine among 213 patients with PCD treated at one center and found that the rate of seropositive response at a median of 20 days after the second vaccine dose was 80.0% and 70.9% for anti-RBD IgG antibodies and NA, respectively.

Although this response was lower than the response observed in immunocompetent HCW (98.9% for both anti-RBD IgG antibodies and NA), it was still higher than the response observed in other immunosuppressed populations. For example, the response rate was 55% in treatment-naïve patients with chronic lymphoid leukemia (CLL) and 16% in CCL patients who were treated at the time of vaccination [10]. Additionally, only 18% of heart transplant recipients [22] and 35% of kidney transplant recipients [23] developed antibodies following the second vaccine dose.

The evaluation of vaccine efficacy through comparison between the rates of infections would not have been informative due to the relatively small numbers of patients with COVID-19 and the different incidence rates in different periods. Given the strong correlation between vaccine efficacy and the production of protective antibodies that has emerged from human vaccine studies [24, 25], it seemed appropriate to rely on this parameter to define the efficacy of the vaccine in different subpopulations at-risk. Moreover, several studies have shown that RBD-IgG concentrations and SARS-CoV-2 neutralizing titers in sera increase with time after the first vaccine dose and more so after the second dose [24, 26]. It has been reported that 14 days after the booster dose, GMT reached 1.9- to 4.6-fold of those measured in COVID-19 convalescent sera [27].

Our results, in a relatively large cohort of patients with PCD confirm the results of other studies and agree well with recent published data [19, 28]. Our findings also indicate that NA activity is evident 3 weeks after the second vaccination; however, NA titers were significantly lower among patients with PCD compared to those of the controls.

Other studies have reported lower humoral response to vaccination among patients with PCD. It has been suggested that the etiology of the lower humoral response is multifactorial, involving both disease-related immune dysregulation and therapy-related immunosuppression [29]. Lockmer et al. [30], who evaluated the antibody response after 2 doses of the BNT162b2 vaccine in 93 MM patients, have found that treatment with anti-B-cell maturation antigen (BCMA), daratumumab and dexamethasone are strongly associated with lower spike protein levels. van Oekelen et al. [19] reported similar findings. Pimpinelli et al. [31] have also reported that treatment with daratumumab has a significant influence on levels of IgG binding or on neutralizing activity. Chung et al. [32] measured anti–SARS-CoV-2 spike IgG titers and neutralizing activity at 1 and 3 months from initial vaccination in 551 hematologic malignancy patients with leukemia, lymphoma, and MM. Patients with lymphoma on observation had the highest median antibody response at 3 months. Patients with MM on maintenance therapy with IMiDs (lenalidomide or pomalidomide) had relatively intact responses, with median antibody titers at 3 months close to those of patients with lymphoma on observation. In contrast, markedly attenuated seroconversion rates and absolute antibody titers were observed in patients treated with BTK inhibitors, venetoclax, PI3K inhibitors, anti-CD19/CD20–directed therapies, and anti-CD38/BCMA–directed therapies.

The COVID-19 pandemic remains a threat to the general population and particularly to immunocompromised populations, including patients with PCD. Compared to the control cohort, the PCD cohort in our study had a significantly greater proportion of patients with underlying chronic pulmonary disease and cardiac disease, placing this group at considerable higher risk of COVID-19 [33, 34]. However, these co-morbidities did not influence humoral response.

In the present study, heavily pretreated patients had significantly lower NA. As not all antibodies against the RBD IgG are neutralizing [35], the NA response is clinically more meaningful than that of anti-RBD-IgG antibodies for preventing infection.

We and others have tried to understand which factors contribute to the observed humoral response in patients with PCD after being vaccinated with the BNT162b2 vaccine. Our analysis has shown that the number of prior therapy lines and age were statistically significant predictors of humoral response in our PCD patient population. In another study conducted in Israel, Herzog-Tzarfati et al. have found that older age, higher lactate dehydrogenase, and number of treatment lines correlated with lower seropositivity likelihood and antibody titers, while absolute lymphocyte count, globulin level, and time from last treatment to vaccination correlated with higher seropositivity likelihood and antibody titers in a cohort of patients with hematologic malignancies [36]. Similarly, Chung et al. [32] have reported that younger patients with hematological malignancies generated higher antibody titers than older patients. Avivi et al. [28] have reported that elderly patients, those with hypogammaglobulinemia and heavily pretreated patients had lower response rates after two vaccine doses. In a national prospective study in Lithuania anti-S1 IgG antibody concentration was strongly correlated with age in patients with hematological malignancies after two vaccinations [20]. Bitoun et al. have reported that the main predictive factor of absence of response was MM disease status [37].

As previously reported [28], the BNT162b2 vaccine has a tolerable safety profile. The rates of local and systemic AEs were lower in the PCD patient population compared to the controls–both after the first and second vaccinations probably due to the lower immunogenic response in immunocompromised patients. No serious AEs were reported. In line with a previous report [38], the second dose was associated with more adverse events than the first dose in immunocompetent individuals [38].

In conclusion, in the current cohort of 213 patients with PCD at different stages of the disease who were treated with various therapy protocols, the humoral response (anti-RBD IgG antibodies and NA levels) was lower than that of the immunocompetent control population, but relatively higher than the humoral response reported in the literature for other immunosuppressed populations. A higher number of prior therapy lines and older age predicted lower humoral immune response in patients with PCD. The frequency of local and systemic AEs reported after vaccination were lower in patients with PCD compared to the control population of HCW suggesting good tolerability of the vaccine. The results support the vaccination protocol in this patient population. Regular monitoring of elderly MM patients for NA and anti-RBD IgG antibody levels, following the second vaccine doses seems prudent in order to identify the optimal time to administer booster vaccinations and minimize the risk for SARS-CoV-2 infection in this susceptible population. Monoclonal antibodies should be offered to patients with low humoral response following vaccination.

## Supporting information

S1 Dataset (XLSX)
